# Phenotypic Screen Identifies a Small Molecule Modulating ERK2 and Promoting Stem Cell Proliferation

**DOI:** 10.3389/fphar.2017.00726

**Published:** 2017-10-24

**Authors:** Chang Yin, Temesgen Fufa, Gayathri Chandrasekar, Madhu Aeluri, Verina Zaky, Shaimaa Abdelhady, Antonio B. Rodríguez, Johan Jakobsson, Farzaneh Shahin Varnoosfaderani, Jayashri Mahalingam, Jianping Liu, Olle Larsson, Outi Hovatta, Frank Gaunitz, Anita Göndör, Michael Andäng, Satish S. Kitambi

**Affiliations:** ^1^Department of Microbiology, Tumor and Cell Biology, Karolinska Institutet, Stockholm, Sweden; ^2^Department of Oncology-Pathology, Karolinska Institutet, Stockholm, Sweden; ^3^Klinik und Poliklinik für Neurochirurgie, Universitätsklinikum Leipzig, Leipzig, Germany; ^4^Department of Biosciences and Nutrition, Karolinska Institutet, Stockholm, Sweden; ^5^Dr. Reddy's Institute of Life Sciences, University of Hyderabad Campus, Hyderabad, India; ^6^Department of Physiology and Pharmacology, Karolinska Institutet, Stockholm, Sweden; ^7^Department of Medical Biochemistry and Biophysics, Karolinska Institutet, Stockholm, Sweden; ^8^The Pirbright Institute, Woking, United Kingdom; ^9^Division of Obstetrics and Gynecology, Department of Clinical Sciences, Intervention and Technology, Karolinska Institutet, Karolinska University Hospital, Stockholm, Sweden

**Keywords:** small molecules, stem cells, phenotype, zebrafish, mouse, PDD

## Abstract

Stem cells display a fundamentally different mechanism of proliferation control when compared to somatic cells. Uncovering these mechanisms would maximize the impact in drug discovery with a higher translational applicability. The unbiased approach used in phenotype-based drug discovery (PDD) programs can offer a unique opportunity to identify such novel biological phenomenon. Here, we describe an integrated phenotypic screening approach, employing a combination of *in vitro* and *in vivo* PDD models to identify a small molecule increasing stem cell proliferation. We demonstrate that a combination of both *in vitro* and *in vivo* screening models improves hit identification and reproducibility of effects across various PDD models. Using cell viability and colony size phenotype measurement we characterize the structure activity relationship of the lead molecule, and identify that the small molecule inhibits phosphorylation of ERK2 and promotes stem cell proliferation. This study demonstrates a PDD approach that employs combinatorial models to identify compounds promoting stem cell proliferation.

## Introduction

Stem cells offer broad biomedical applicability and display fundamentally different mechanism of proliferation (Andang et al., 2008; Johansson et al., 2010; Yang et al., 2011) and differentiation (Andang et al., 2008; Theofilopoulos et al., 2013, 2014) control when compared to somatic cells. This denotes that their tremendous potential can be better realized by understanding such mechanisms. The unbiased approach used in Phenotype based drug discovery (PDD) programs can offer a unique opportunity to identify such novel biological phenomenon.

Phenotypic drug discovery (PDD) screening is re-emerging as an alternative platform of drug discovery (Swinney and Anthony, 2011; Lee et al., 2012; Sams-Dodd, 2013) and offers multiple advantages over target-based screening (Sams-Dodd, 2005; Lee and Berg, 2013). Various cell based and small animal models have been developed and successfully employed for PDD screening and various lead molecules have been identified (Szabo et al., 2017). However, the models employed and the robustness of the phenotypes dictates the scalability of screening and reliable validation of identified hits. In addition, structure-activity relationship and target deconvolution studies are relatively slow in a PDD setup (Szabo et al., 2017). The salient feature associated with phenotypic screening approach is that it evaluates observable phenotypic changes in a cell, tissue or an entire organism irrespective of the underlying mechanism (Lee et al., 2012). This approach has higher biomedical relevance since physiological response, cell to cell/tissue interaction or crosstalk across multiple signaling mechanisms participate in producing the phenotype (Lee et al., 2012). Therefore, to maximize relevant lead identification with an increased therapeutic applicability, a PDD screening setup against a well-defined phenotype is absolutely necessary. For a successful PDD program, phenotype validation across complementary models and its *in vivo* translation is of utmost necessity. Hence, to minimize false positives and maximize biomedical relevance, a combinatorial screening approach is required and would be beneficial.

Stem cells are a promising model for screening, discovery and development of drugs (Kitambi and Chandrasekar, 2011). Given their potential therapeutic applications, various stem cell PDD platforms have been developed and used in drug discovery and toxicity studies. However, stem cells from different tissues are not the same. In addition, there are limitations with regard to their expandability, hindering large scale PDD screens. Embryonic stem cells (ESC) offer a powerful tool to conduct PDD screens and could have a major impact on drug development and toxicity studies. For a successful PDD on ESCs, screening against a properly defined phenotype and its reproducibility across various PDD screening platforms is necessary. Here, we perform a PDD screen measuring colony size phenotype of mouse and human embryonic stem cells as a readout. This phenotype based screen allows for a straightforward and rapid assessment of effects produced by compounds.

In this study, we conduct a combinatorial phenotypic screening using mouse and human embryonic stem cells and a transgenic zebrafish model to identify one compound increasing stem cell proliferation. The combinatorial use of *in vitro* and *in vivo* approaches allows reliable validation of the phenotype and assesses the efficacy of the identified hit. We also use the phenotypic approach to characterize the immediate effects produced by the small molecule, its structure-activity relationship, its mechanism of action and we explore its biomedical applicability. This work demonstrates the strength of a broad combinatorial phenotypic screening approach in phenotype identification, lead generation and validation and its biomedical applicability.

## Materials and methods

### Cell culture

Human embryonic stem cells (hESCs) were grown in dishes coated with Matrigel (BD Biosciences). mTesR1 medium was used for hESC cell line HS181 culture (Fu et al., 2011; Rodin et al., 2014). Confluent cells were split 1:3–5 after trypsinization using TrypLE Express (Invitrogen). Mouse R1 embryonic stem cells (mESCs) were cultured as suspension in dishes, in DMEM/F12 containing 0.4 mM 2-mercaptoethanol, 5 mM HEPES and N2 supplement (all from Invitrogen), 1,000 U/ml leukemia inhibitory factor (LIF) and 10 ng/mL basic fibroblast growth factor (FGF) (Chemicon) as described previously (Andang et al., 2008). Adult mouse neural stem cells (mNSCs) were isolated from the lateral ventricle dissected from the brain of C57BL6 mice and cultured in DMEM medium containing Glutamax medium at a final concentration of 2 μM (Invitrogen, USA), FGF-2 (20 ng/ml), B27, and EGF (20 ng/ml for each) as previously described (Wachs et al., 2003). Human foreskin fibroblast cells were grown in DMEM medium with 10% fetal bovine serum (all from Invitrogen).

Human embryoid bodies (EB) were generated as previously described (Fu et al., 2011; Rodin et al., 2014). HS181 hESC cells were grown on laminin-521 coated 6 well plates (Sarstedt) with each well containing 3,000 cells. Knockout DMEM supplemented with 2 mM L-glutamine, 20% fetal calf serum, 0.1 mM β-mercaptoethanol and 1% non-essential amino acids (all from GIBCO) was used to culture cells. EB's were obtained after 1–2 weeks of culture.

### Small molecules

The NCI Diversity Set II small molecule library containing 1364 small molecules was analyzed *in silico* using JChem for Excel (ChemAxon) for clustering based on amenable chemistry and structural compatibility for biological testing. The identified compounds were obtained as 10 mM DMSO stock solution from the NCI/DTP Open Chemical Repository (http://dtp.nci.nih.gov/) and used in the subsequent screening.

The identified lead compound and compounds that were structurally similar to the lead were purchased from Enamine (www.enamine.net). Small molecule Ulixertinib was purchased from Tocris Biosciences. All compounds were dissolved in DMSO to a stock concentration of 10 mM.

### Small molecule screening setup and hit selection

Three parallel PDD screens were undertaken on mESCs, hESCs, and islet1:GFP transgenic zebrafish using the NCI Diversity Set II small molecule library.

#### mESC screen and hit selection

Clear-bottom 96-well microtiter plates (Corning) were coated with 0.2% gelatin (Sigma) 3 h prior to use for primary screening with mESCs. Cells were diluted and seeded at a density of 10,000 cells per well in 100 μl of medium. The compounds at final concentration of 5 μM were added into each well after seeding. Cells were cultivated for 4 days allowing the formation of colonies. Outer wells were not used for screening, but served as control wells. Bright-field images of colonies were acquired after 4 days of culture with or without compound. A simple phenotypic screening assessing mESC colony sphere size was used as endpoint to categorize compound effects.

#### hESC screen and hit selection

Human ESC cells were cultured (10,000 cells per well in 100 μl of medium) as above in 96 well plates and exposed to 87 compounds (to a final concentration of 5 μM) identified from mESC screen. Cell viability was measured post 10 days of treatment with compound. A total of 33 compounds were identified in the hESC primary screen. These 33 compounds were then subjected to rescreening and top five compounds were selected. These top five hits (including D1) produced an increase in ATP of mESC and hESC cells.

#### Compound treatment of human embryoid bodies

To perform compound treatment on human embryoid bodies (EBs), HS181 cell were plated in a 6-well plate with each well containing 3,000 cells. To the EB culture medium (as described above) DMSO or 0.05 μM D1 was added. Culture medium with or without compound was changed every second day and the treatment was performed for 11 days. Post 11 days of culture, the generated EBs were photographed, fixed and taken for immunostaining as previously described (Rodin et al., 2014).

#### Zebrafish screening

A zebrafish-based screening platform was used using the *CellIQ* imaging system. Transgenic zebrafish embryos *Tg(isl1:GFP)*, with GFP expression controlled by the islet1 promoter, were obtained by natural mating and collected in egg water with 0.03% PTU (*N*-Phenylthiourea) to inhibit pigment formation. Three to five embryos were distributed into the wells of a 96-well plate with 100 μl of PTU treated egg water. Compounds (final concentration 10 μM) were added into the corresponding wells for 48 h incubation. Each well was photographed 48 h post-fertilization (hpf) using the built-in setup in *CellIQ*. Fluorescence of GFP in each well was quantified and the signal intensities were compared with those of control wells treated with DMSO. Compounds inducing a higher GFP fluorescence were plotted using Microsoft excel.

A majority of compounds did not produce any observable effects, 1% of the compounds caused developmental delay and 0.2% were lethal to the embryo (Supplementary Figure 1D). Quantification of GFP intensity was done to identify six compounds that showed an increase in intensity without producing any developmental defects on the zebrafish embryos (Supplementary Figure 1E). One compound out of the six (D1) that produced an increase in both mESC colony size and hESC cell viability was selected as hit. The hit compound D1 produced an increase in colony size and viability of mESC and hESC and increased GFP signal in zebrafish.

### Kinome screen

The protein kinase siRNA library (SMARTpool, Cat. No. G-003505-E2-01, 719 genes in three 384-well plates) was purchased from Thermo Scientific (Sweden) and diluted with siRNA buffer (# B-002000-UB-100, GE LifeScience) to 400 nM. Replicates of the library were prepared as ready-for-transfection (5 μl/well of 400 nM siRNA) using Biomek FX^p^ Liquid Handling Automation Workstation (Beckman Coulter, Sweden) and frozen till use. BD falcon 384-well black plates with clear bottom (Cat. No. 353962, VWR, Sweden) was used for screening. Before transfection, the plates were thawed to room temperature, a reverse transfection mix of 5 μl/well of Opti-MEM reduced serum medium (Cat. No. 31985062, Thermo Scientific) containing 0.5 μl HiperFect transfection reagent (Cat. No. 301704, Qiagen) was added into the siRNA plates containing 5 μl of 400 nM sample or control siRNAs. After brief shaking, the plates were incubated at room temperature for about 10–15 min. On top of the siRNA-HiPerFect complex, 10,000 mESC cells in 40 μl were added (40 nM siRNA). The cells were cultured for 12 h prior at 37°C. Post-incubation, 50% of the media was carefully removed from the top portion of each well and replaced with fresh mESC media. After a total 48 h of incubation, 50% of media was carefully removed from the top portion of each well and replaced with fresh media containing DMSO or 0.05 μM D1. The plates were then incubated for 48 h and then taken for cell viability measurement.

Post-viability measurement, the values obtained from D1 treated cells were subtracted from DMSO treated cells to obtain relative change of values. The relative change of viability in D1 treated cells post-siRNA knockdown of all kinases values were plotted using Prism Software.

### Cell viability and cytotoxicity studies

Influence on cell viability and cytotoxicity was determined by using mESCs or hESCs cultivated in 384-well microtiter plates at a density of 10,000 cells per well in 45 μl of growth medium. Then, an appropriate concentration of a compound to be tested was added to the wells (5 μl) and the cells were incubated for four days (mESCs) or for 4 or 10 days (hESCs). Cell viability was determined using the CellTiter-Glo® Luminescent Cell Viability Assay (Promega) measuring the total amount of ATP in cell lysates. In addition, the CytoTox-Glo Cytotoxicity Assay (Promega) was employed to determine the release of lactate dehydrogenase from the cells as a measure of a necrotic loss of membrane integrity. Triplicate or quadruplicates were used to assess the effect produced and standard deviation (STD) was used to measure the statistical significance.

For the determination of the dose-response effect, a 96-well compound plate (TPP, 92097) was prepared for a serial dilution of each compound from 10 mM to 0.17 mM in 100% DMSO in columns 1–11. Negative (100% DMSO) and positive (Staurosporin) controls were placed in rows A–D and E–H, respectively, of column 12. The compounds (10 μl) were subsequently diluted with 190 μl of growth medium and 5 μl compound solution of each dilution was transferred to quadruplicate wells of a sterile 384-well black clear bottom plate (BD Falcon) containing cells in 45 μl of growth medium. The plate was incubated for 24 or 72 h, followed by luminescence measurements using a Victor3 (Perkin Elmer) microplate reader. Total luminescence was normalized to the DMSO negative controls and curve fitting was performed using GraphPad Prism (v6.02).

For phenotype-based assessment of structurally similar compounds, a dilution series was performed in a 96-well plate using mESCs. Cells were incubated for 4 days prior to imaging and the colony size was calculated using ImageJ. The values were then used to generate a heatmap representing mESC colony size. Triplicate or quadruplicates were used to assess the effect produced and standard deviation (STD) was used to measure the statistical significance.

### Flow cytomentry based analysis

For Flow cytometry-based cell cycle profiling, mESCs were incubated for 2 or 4 days with either DMSO or compound at the concentrations indicated. Then, cells were pulsed 20 min with EdU or BrdU, followed by dissociation and resuspension in 1 ml PBS. Then, cells were fixed with 75% ethanol overnight and rehydrated in PBS, followed by EdU or BrdU staining and propidium iodide (PI) (Roche) staining as described previously (Andang et al., 2008).

The percentage of apoptotic and dead mESCs was quantified by flow cytometry after double staining with Annexin V and propidium iodide (PI) (Roche). In brief, after treatment with DMSO, compound or staurosporin, mESCs were trypsinized, suspended in 100 μl incubation buffer containing 2 μl Annexin V and 2 μl PI supplied in the kit, and kept in the dark for 10 min at room temperature. The cells were analyzed within 1 h. Flow cytometry was performed using a FACScan instrument and CellQuest Pro. Triplicate were used to assess the effect produced and standard deviation (STD) was used to measure the statistical significance.

Final analysis was done using FlowJo software (Tree Star, Ashland, OR, USA).

### Cell immunohistochemistry

The cells were permeabilized with PBS containing 0.1% Triton X-100 and blocking was performed with PBS containing 5% BSA. Immunostaining of mESCs or mNSCs was performed using anti-Oct4 (Millipore), anti-SSEA (Millipore), anti-Nestin (abcam), anti-GFAP (abcam), anti-active cleaved caspase 3 (abcam) for mouse mESCs or NSCs and anti-Nanog (Cell Signaling), anti-Oct4 (Millipore), anti-sox2 (Cell Signaling), anti-nestin (Cell Signaling) and anti-GFAP (abcam) for hESCs. Analysis by microscopy was performed after costaining with DAPI.

### Cell extracts and western blotting

Cell extracts were obtained by using RIPA buffer (ThermoFisher Scientific). Samples were analyzed by western blotting with the following antibodies: anti-nucleolin (abcam), anti-sox2 (Millipore), anti-oct4 (Millipore), anti-p44/p42 ERK1/2 (Cell Signaling), anti-phospho-p44/p42 ERK1/2 (Cell Signaling) and anti-β-actin (Millipore) using antibody dilutions as recommended by the manufacturer. Triplicate were used to assess the effect produced and standard deviation (STD) was used to measure the statistical significance.

### RNA isolation and cDNA synthesis

Total RNA from DMSO or compound treated mESCs was isolated using an RNA isolation kit (RNeasy Mini Kit, Qiagen) according to manufacturer's protocol. Three hundred nanograms of total RNA was used for cDNA synthesis using SuperScript III First strand synthesis and SuperMix (Invitrogen) was used for qRT-PCR according to manufacturer's protocol.

### Quantitative PCR

Quantitative PCR was performed using the SYBR® Select Master Mix kit (Applied Bioscience) and the primers as listed in Table 1. Samples were analyzed on a Rotor-Gene 6000 and data was obtained by the Corbett research series software 1.7. Runs were performed with initial 2 min heat activation at 95°C followed by 40 cycles of 30 s at 95°C, 30 s at 60°C and 60 s at 72°C. Relative expression levels and significance were determined using the ΔΔCt method and presented after analysis by GraphPad Prism 6. Triplicate were used to assess the effect produced and standard deviation (STD) was used to measure the statistical significance.

**Table 1:** qPCR primers designed against mouse target genes.

**Gene**	**Forward primer 5′–3′**	**Reverse primer 5′–3′**	**Marker for**
oct 4	GCTCTCCCATGCATTCAAAC	TGTCTACCTCCCTTGCCTTG	Pluripotency
Sox2	ATGGCCCAGCACTACCAGAG	CTTCTCCAGTTCGCAGTCCA	Pluripotency
Klf4	CAGTGCCAGAAGTGTGACAGG	TCGTGGGAAGACAGTGTGAA	Pluripotency
Nanog	TTTGGAAGCCACTAGGGAAAG	AAGCCCAGATGTTGCGTAAGT	Pluripotency
Fgf5	ACTGAAAAGACAGGCCGAGA	TGAACCTGGGTAGGAAGTGG	Primitive Ectoderm
Gsc	AAAGCCTCGCCGGAGAA	AGCTGTCCGAGTCCAAATCG	Epiblast
Lhx1	CACCTCAACTGCTTCACCTG	TGTTCTCTTTGGCGACACTG	Mesoderm
Wnt3	CAGCGTAGCAGAAGGTGTGA	GCCAGGCTGTCATCTATGGT	Mesoderm
Fgf8	CACAGAGATCGTGCTGGAGA	TGTACCAGCCCTCGTACTTG	Mesoderm
Sox17	CCGAGATGGGTCTTCCCTAC	CGTCAAATGTCGGGGTAGTT	Endoderm
Sox1	CACAACTCGGAGATCAGCAA	CTCGGACATGACCTTCCACT	Ectoderm
Gata6	GAACGTACCACCACCACCAT	CCATGTAGGGCGAGTAGGTC	Endoderm
Bmp2	GCTCCACAAACGAGAAAAGC	AGCAAGGGGAAAAGGACACT	Endoderm
GAPDH	GAG AAA CCT GCC AAG TAT GAT GA	AGA CAA CCT GGT CCT CAG TGT A	

### Animal maintenance and tissue collection

All animal work was performed in accordance with the national guidelines and after approval by the local ethical committee Stockholm Norra Djurförsöksetisks.

For mice experiments, wildtype mice were housed spaciously and experiments were carried out according to the approved protocols. Perfusion and fixation were performed as previously described (Deferrari et al., 2003; Phiel et al., 2003).

Wild type or transgenic zebrafish were maintained at 28.5°C under standard conditions of light/dark cycle, feeding, care and egg collection. Embryos were collected in egg water after natural mating and staged according to Kimmel et al. (1995). Embryos were staged in hours post-fertilization (hpf) and days post-fertilization (dpf). The collected embryos were first anesthetized using 0.1% Tricane, kept on ice and fixed at different stages in 4% PFA overnight, then washed with PBS containing 0.1% Tween-20 (PBSTw).

### Compound treatment in zebrafish

Zebrafish embryos were collected and distributed in a 6 well plate with each well containing 10 embryos. Embryos were then incubated with 2 ml of egg water with or without compound. DMSO was added as control and D1 was tested at two different concentrations (10 and 15 μM). The embryos were incubated for 2 or 3 days after which they were anesthetized using Tricane on ice and photographed.

### Mouse neural stem cell isolation and culture

Neural stem cells from adult C57BL6 male mice were isolated according to previously published protocols (Wachs et al., 2003; Sievertzon et al., 2005). Equal amounts of cells isolated from the lateral ventricle were distributed in 12 well culture dishes (Corning) and treated with 0.05 μM D1 or DMSO. The cultures were allowed to grow for 4 days after which the spheres obtained were counted, fixed and used for immunostaining.

## Results

### Identification of a lead compound using combinatorial models based screening

**Figure 1:**
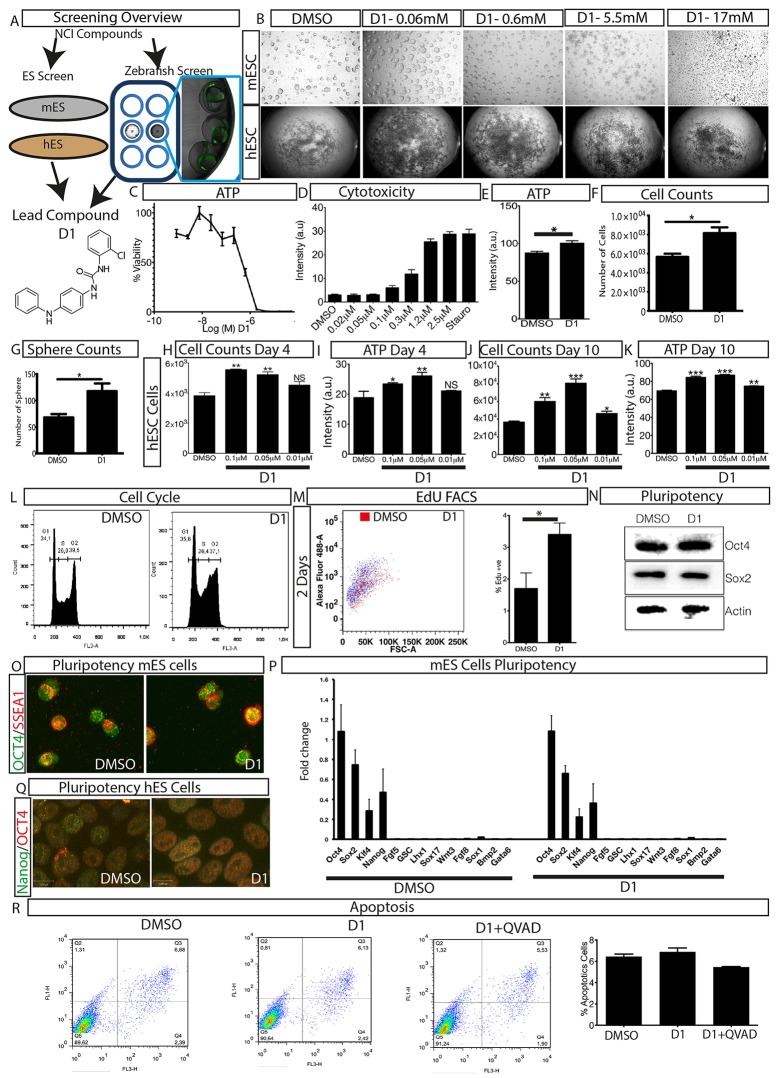
Overview of phenotypic screen and hit characterization**. (A)** Phenotypic screening overview showing two parallel screens performed on embryonic stem cells (mouse mES, human hES) and zebrafish, and identification of lead compound D1. **(B)** Effect on colony size after treatment with different concentrations of compound D1 on embryonic stem cells. **(C)** Cell viability assay of D1 measuring ATP levels of mESCs after 4-day treatment with different concentrations. **(D)** Cytotoxicity of D1 on mESCs at different concentrations after 4-day incubation. **(E)** Cell viability assay measuring ATP after 4-day treatment of mESCs with 0.05 μM D1. **(F)** Cell count after 4-day treatment of mESCs with 0.05 μM D1. **(G)** Number of mESC colony spheres obtained after 4 days of treatment with DMSO or 0.05 μM D1. **(H–K)** Number of cells obtained and the effect on ATP after treating hESCs for 4 and 10 days with DMSO or different concentrations of D1. **(L)** Flow cytometry based cell cycle analysis after 4-day treatment of mESCs with 0.05 μM D1. **(M)** Flow cytometry based quantification of EdU positive cells after 2-day treatment of mESCs with 0.05 μM D1. **(N,O)** Assessment of pluripotency of mESCs using western blotting **(N)** and immunostaining **(O)** after 4-day treatment with 0.05 μM D1. **(P)** Quantitative PCR based measurement of various pluripotency and differentiation markers of mESCs treated with DMSO or 0.05 μM D1. **(Q)** Immunostaining of hESCs to assess the expression of pluripotency markers. **(R)** Quantification of apoptosis using Annexin V staining after 4-day treatment of mESC with 0.05 μM D1. mESC, mouse embryonic stem cells; hESC, human embryonic stem cells. Data represent mean ± STD, ^*^P < 0.05, ^**^P < 0.01, ^***^P < 0.001 compared to control treatment.

Three parallel models were employed to conduct phenotypic screening in order to identify the lead compound 1-(4-anilinophenyl)-3-(2-chlorophenyl) urea (PubChem substance ID SID26664806) abbreviated as D1 (Figure 1A, Supplementary Figures 1A–E, 2A). Mouse embryonic stem cells (mESCs) were grown as suspension cultures and exposed to 1,364 different small molecules from the NCI Diversity set II library at 5 μM concentration for 4 days. A simple phenotypic screening assessing mESC colony sphere size was used as endpoint to categorize compound effects. DMSO was used as solvent for the compounds here and treatment with similar concentration of DMSO produced a phenotype similar to untreated control cells. Our screening showed that 62.6% of the compounds produced a colony size similar to that seen with DMSO treatment, which was classified as control (Supplementary Figure 1A). Eighteen percent of the compounds were lethal and classified as phenotype 1, 13% of the compounds produced smaller mESC colonies and were categorized as phenotype 2. A mixture of large and small colonies, categorized as phenotype 3, was produced by 2.4% of the compounds (Supplementary Figure 1A). The screen also identified 4% of the compounds, including D1, to increase colony size (Supplementary Figure 1A), grouped as phenotype 4. A total of 87 compounds, producing phenotype 3 and 4 (Supplementary Figure 1A, Supplementary Table 1), were selected for further screening to assess the cell viability of human embryonic stem cells (hESCs) (Supplementary Figures 1B,C, Supplementary Table 1). Cell viability screening and rescreening identified five compounds, including D1, which consistently produced an increase in ATP (Supplementary Figures 1B,C). In parallel to the mESC and hESC based screening, a phenotypic screening using zebrafish embryos expressing green fluorescence protein (GFP) under the control of the Islet1 promoter was performed. Transgenic zebrafish embryos at one cell stage were exposed to compounds at 10 μM and their effects on embryo development and GFP intensity were scored using bright field and fluorescent imaging. The screen identified that the majority of compounds did not produce any observable effects, 1% of the compounds caused developmental delay and 0.2% were lethal to the embryo (Supplementary Figure 1E). Quantification of GFP intensity identified six compounds that showed an increase in intensity without producing any developmental defects on the zebrafish embryos (Supplementary Figure 1F). One compound (D1) out of the six also that produced an increase in mESC colony size and hESC cell viability while not producing any effect on fibroblast cells (Supplementary Figure 1D). Thus, using a combination of *in vitro* and *in vivo* phenotypic screening we identified D1 that increased mESC colony size, hESC cell viability and produced an increase in GFP expression in zebrafish embryos.

### D1 causes increase in proliferation *in vitro*

In order to understand the phenotype produced by D1, a dilution series experiment was performed on mESCs and hESCs and its effect on colony size was assessed. Exposure to D1 produced large colonies in both *in vitro* models at lower concentrations (Figure 1B). The colony size dramatically decreased with increased concentrations of D1 indicating a cytotoxic effect (Figure 1B). The phenotype produced on mESC colony size was also reflected by cell viability and cytotoxicity measurements, in which lower concentrations of D1 were shown to increase cell viability and to cause less cytotoxicity (Figures 1C,D). Based on these results, further *in vitro* evaluation on mESC was done with 0.05 μM D1, unless otherwise specified. Exposure to D1 increased mESC colony sphere size and sphere number (Figure 1G, Supplementary Figure 2A), cell viability (Figure 1E) and number (Figure 1F), produced a broader G2 phase (Figure 1L, Supplementary Figure 2B) with increased EdU or BrdU labeling at 2 days (Figure 1M) and 4 days of culture (Supplementary Figure 2C) indicating more cells in the S phase of the cell cycle. Exposure of hESC culture to 0.1, 0.05, and 0.01 μM D1 was done and its effect on cell number and ATP levels was measured at 4 and 10 days of culture (Figures 1H–K). An increase in cell number and ATP levels was seen at both 0.05 and 0.1 μM D1 treatments for 4 and 10 days, however, 0.01 μM D1 treatment resulted in a significant increase only after 10-day culture, suggesting that treatment with lower concentrations requires a longer period of exposure to produce an effect (Figures 1H–K). In conclusion, the results demonstrated that D1 treatment produced more cells, increased colony size of both mESC and hESC cells.

### Effect of D1 on pluripotency

To examine the effect on pluripotency, mESCs and hESCs were exposed to D1 for 4 and 10 days, respectively, and pluripotency was assessed using various markers (Figures 1N–Q, Supplementary Figures 2D,E). Western blotting of mESC extracts and immunostaining with pluripotency markers on mESCs and hESCs did not show any difference between treatment and controls (Figures 1N–Q, Supplementary Figure 2D). Quantitative PCR with RNA isolated from mESCs using primers for various pluripotency and differentiation markers (Table 1) revealed that D1 treatment neither produced any changes in pluripotency nor induced expression of differentiation markers (Figure 1P). Immunostaining on neurospheres obtained from lateral ventricle culture of mouse brain also did not produce any change in pluripotency marker expression (Supplementary Figure 2E). These results indicated that D1 treatment allowed cells to maintain their pluripotent state and does not promote expression of differentiation markers in mESC cultures.

### *In vitro* evaluation of the effects of D1

Quantification of apoptotic cells was done to examine whether the increase in proliferation of mESCs was due to decrease or suppression of apoptosis. A FACS based quantification of apoptotic cells did not show any significant change in apoptotic cells (Figure 1R) indicating that D1 did not promote or suppress apoptosis of mESCs. This was also confirmed by immunostaining for active cleaved caspase 3 in mESCs cultured with or without D1 (Supplementary Figure 2F). Compound D1 did not increase the number of apoptotic cells and measurement of relative mRNA and protein expression of various pluripotency markers did not show any significant change, suggesting that the stemness of D1 treated cells was similar to that of untreated cells in both mESC and hESC cultures (Figures 1N–R, Supplementary Figures 2D–F). Treatment with D1 on hESC cells did not alter embryoid body generation indicating that the compound did not interfere with the differentiation process (Supplementary Figures 2G,H). These results demonstrate a phenotypic screening approach for the identification of a small molecule that causes stem cell proliferation without compromising its pluripotency or differentiation potential.

### Phenotypic assessment of structure activity relationship (SAR) of D1

**Figure 2:**
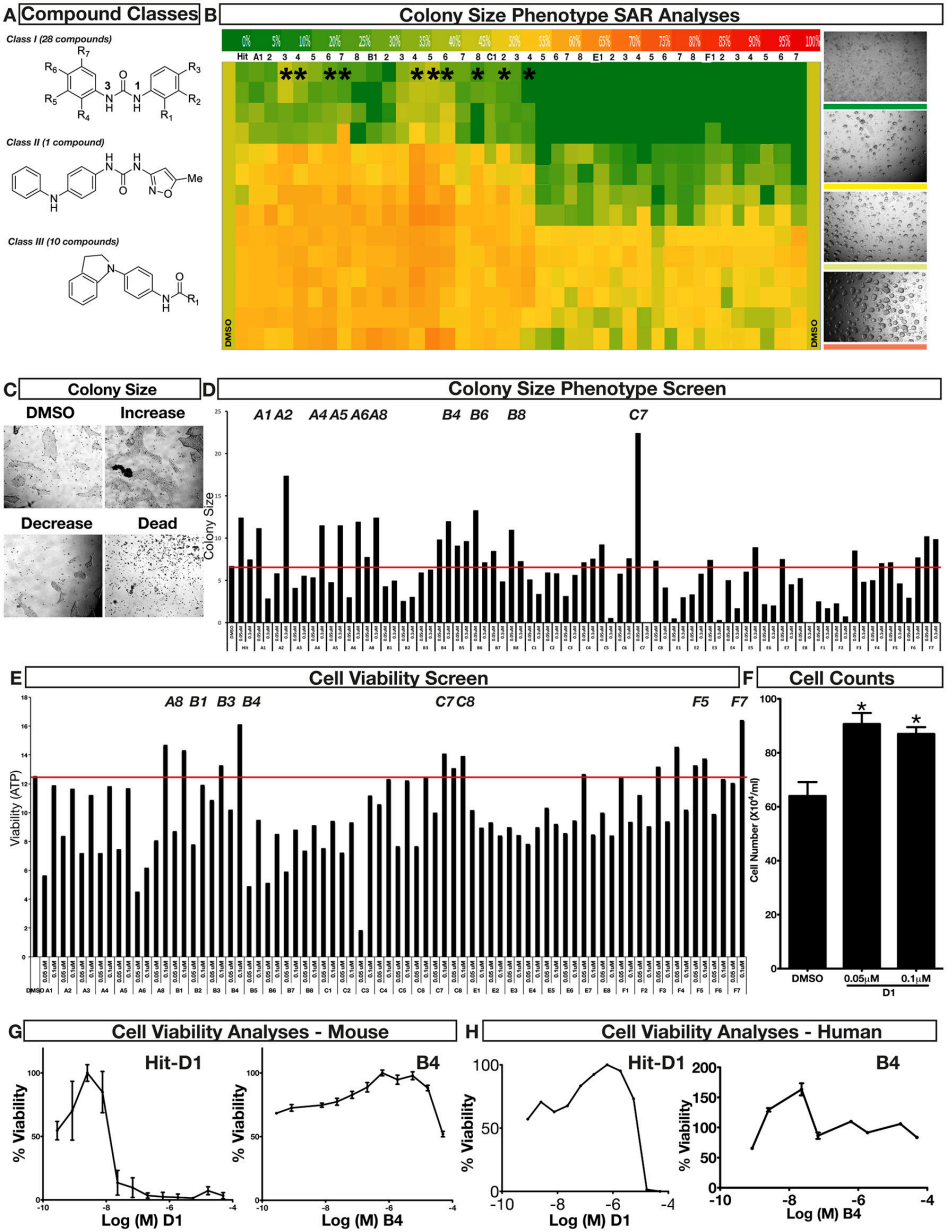
Structure-Activity Relationship (SAR) analysis of compounds related to D1. **(A)** Displayed are the three classes of generic structures investigated with modified positions at R1-R7, representing a total of 39 compounds. **(B)** The heatmap represents the response of ESCs exposed to the 39 compounds (labeled “A1-8, B1-8, C1-8, E1-8, and F1-7”) for 4 days with regard to colony size in comparison to the effect of D1 (signified as hit) and to cells treated with DMSO as control. Each compound was tested using a log dilution series with the highest concentration of 50 μM shown at the top, and the colony size measurement was used to generate a heatmap. Representative images with matching color codes representing the heatmap are shown on the right. Compounds showing an increase in colony size significantly different to cells treated with DMSO are labeled by asterisks. **(C)** Representative images showing hESC colony spread of DMSO treated control and compound treatment producing increase, decrease or lethal effect. **(D)** Measurement of colony size of hESCs post 4-day treatment of the 39 compounds tested in mESCs. The most prominent hits are labeled. **(E)** Cell viability of mESCs after 4-day treatment with two different concentrations of the compounds. The red line represents the baseline control (cells treated with DMSO) and compounds producing an increase in viability are labeled. **(F)** The number of mESCs after 4-day treatment with two different concentrations of D1 (0.05 μM and 0.1 μM). **(G,H)** Cell viability measurement of mESCs **(G)** and hESCs **(H)** after 4-day exposure to Hit- D1 and B4 compounds. Labeling of SAR compounds with D series is avoided in order to prevent misunderstanding of effect produced by hit D1. mESCs, mouse embryonic stem cells; hESCs, human embryonic stem cells; Data represent mean ± std, ^*^P < 0.05, ^**^P < 0.01 compared to control treatment.

To improve our understanding of D1, a structure activity relationship (SAR) studies was performed by measuring colony size. The SAR studies were conducted using a total of 39 structurally similar commercially available urea derivatives (Table 2). A total of 28 compounds grouped into Class I was characterized by *N,N*′-diphenyl substitution, one compound where a phenyl ring in Class I was replaced with an oxazole ring was characterized as Class II (Figure 2A, Table 2). A total of 10 compounds where one of the phenyl rings in Class I was substituted with aliphatic cyclic or acyclic alkyl groups was characterized as class III (Figure 2A, Table 2). The effects of the compounds on mESC and hESC colony size was measured (Figures 2B–D) and filtered using cell viability measurements done on mESC cells (Figure 2E). The SAR analysis identified that 9 compounds belonging to class I (Figures 2B–D) and one compound belonging to class III, caused a significant increase in the mESC and hESC colony size (Figures 2B–D). Phenotypic assessment showed that substitution of a phenyl ring with aliphatic cyclic or acyclic alkyl groups or oxazole renders the compounds to be more toxic (Figures 2B–D). The data from compounds A3-A6 demonstrated that replacing Cl at R1 by H or F, substitution at R2 with Me and at R3 with F or Cl has little or no effect on activity when compared to D1. When phenylamine at R6 was replaced with 4-tetrahydropyranylamine or acetylamine as seen in B4, B5, B6, an increase in colony size over a broad range of concentrations was observed. However, when substitutions were made at R1 with H, at R3 with sulfonamide and at R6 with piperidine or nitro groups as in B1, B2, B3 or when the hydrogen atom present on nitrogen of 4-phenylamino (R6) was substituted with isopropyl as in B7 to C2, an increase in toxicity and a decrease in colony size was observed at higher concentrations (Figures 2B–D, Table 2). Compounds E6-E8 and F1-F7, which were obtained by replacing the 4-phenylamino group (R6) of D1 with hydrogen or bromine and substituting R4, R5, and R7 with various groups such as halo, alkyl, ester, alkoxyl or heterocycles, showed an increase in colony size over a narrow range of concentrations with higher concentrations being toxic (Figures 2B–D, Table 2). Compounds belonging to class II and III showed an overall decrease in colony size (Figures 2B–D, Table 2). Cell viability screen on mESC cells (Figure 2E, Table 2) using two concentrations identified compound B4 as the only compound, in addition to D1, that increases colony size in mESC and hESC and produce increase in viability of mESC (Figures 2B–E, Table 2). Similar to hit D1 which increase mESC number (Figure 2F) and produce increase in cell viability (Figures 2G,H), compound B4, where phenylamino ring at R6 is replaced with 4-tetrahydropyranamino group shows increased the colony size and cell viability of mESC and hESC (Figures 2G,H). This phenotypic approach demonstrated that structural modifications such as the substitution of the phenylamine group (R6) with indoline, nitro or piperidine and replacing the *N*-phenyl ring with cyclic or acyclic alkyl groups as seen in class I, II and III resulted in smaller or no colonies when compared to substitution of phenylamine (R6) with hydrogen or halo and substituting R4, R5&R7 with other groups such as halo, alkyl, ester, alkoxyl or heterocycles, which resulted in a reduction of toxicity. These results identified that phenylamine (R6) and Cl (R1) groups on D1 are crucial and replacing phenylamine (R6) with tetrahydropyranylamine and acetylamine (compounds B4–B6) would lead to decreased toxicity and an increase in viability in a concentration dependent manner.

**Table 2:** Structurally similar compounds.

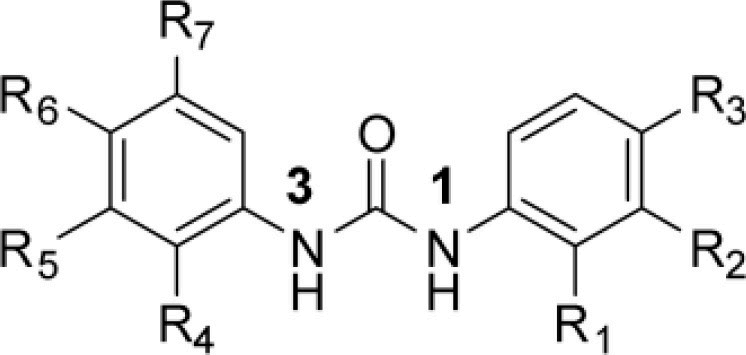								
**S.No**.	**Compound code**	**R1**	**R2**	**R3**	**R4**	**R5**	**R6**	**R7**
1	A1	H	Cl	H	H	H	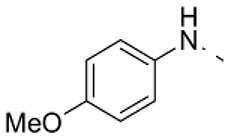	H
2	A2	H	CF3	H	H	H	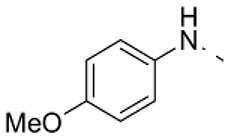	H
3	A3	H	H	Cl	H	H		H
4	A4	F	H	H	H	H	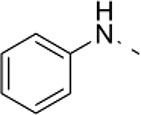	H
5	A5	H	H	F	H	H	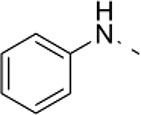	H
6	A6	H	Me	H	H	H	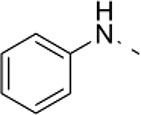	H
7	A7 (HIT-D1)	Cl	H	H	H	H	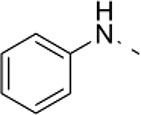	H
8	A8	H	H	OMe	H	H	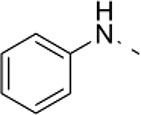	H
9	B1	H	H	SO_2_NH_2_	H	H	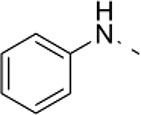	H
10	B2	H	H	NO_2_	Cl	H	H	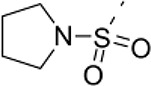
11	B3	Cl	H	H	H	H	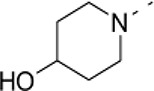	H
12	B4	Cl	H	H	H	H	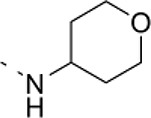	H
13	B5	Cl	H	Me	H	H	NHAc	H
14	B6	Cl	H	F	H	H	NHAc	H
15	B7	H	H	H	H	H		H
16	B8	H	H	F	H	H		H
17	C1	H	CF_3_	H	H	H		H
18	C2	H	Cl	H	H	H		H
19	E6	Cl	H	H	COOMe	H	Br	H
20	E7	Cl	H	H	Me	H	Br	H
21	E8	Cl	H	H	COOMe	H	H	H
22	F1	Cl	H	H	H	OMe	OMe	OMe
23	F2	Cl	H	H	H	H	CF3	H
24	F3	Cl	H	H	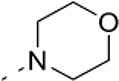	H	CF3	H
25	F4	Cl	H	H	Cl	H	H	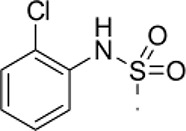
26	F5	Cl	H	H	H	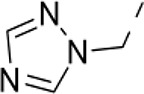	H	H
27	F6	Cl	H	H	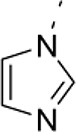	H	CF3	H
28	F7	Cl	H	H	H	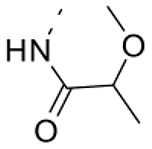		
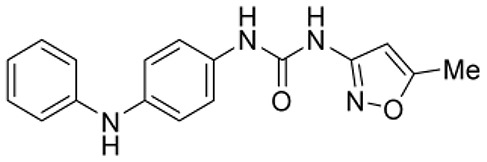								
**C3**								
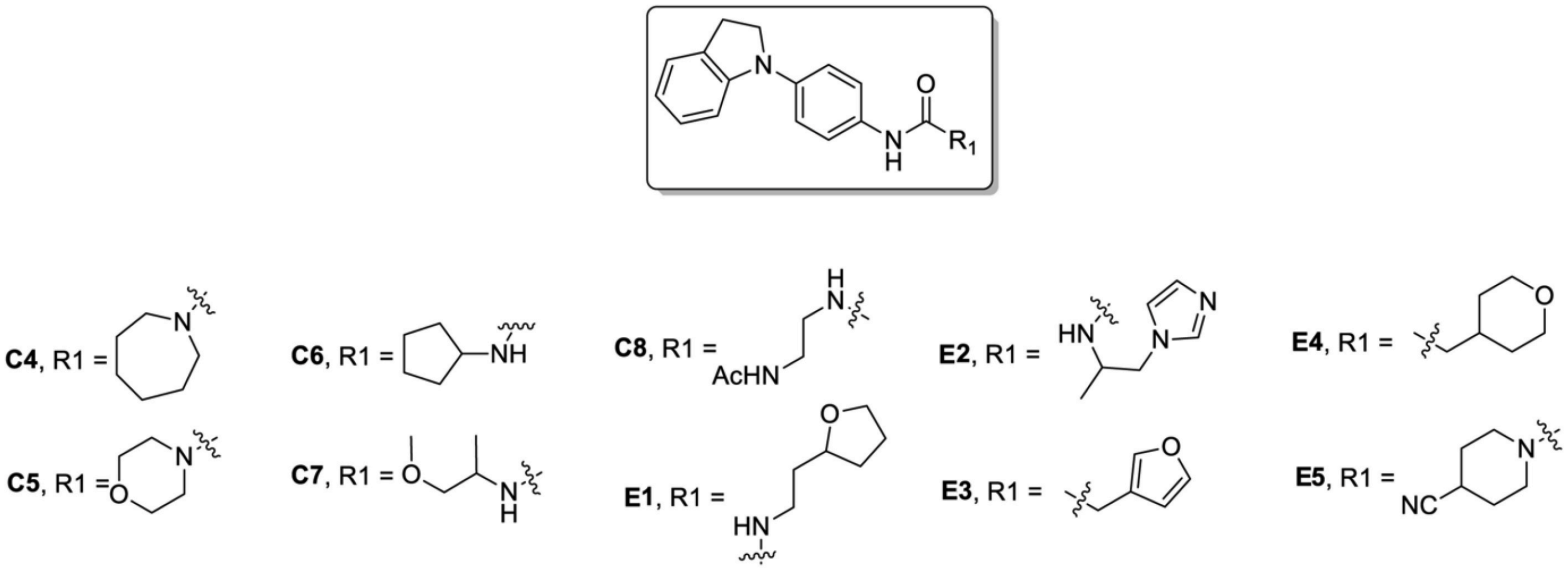								

### Evaluation of the effects of D1 with primary cultures and *in vivo*

**Figure 3:**
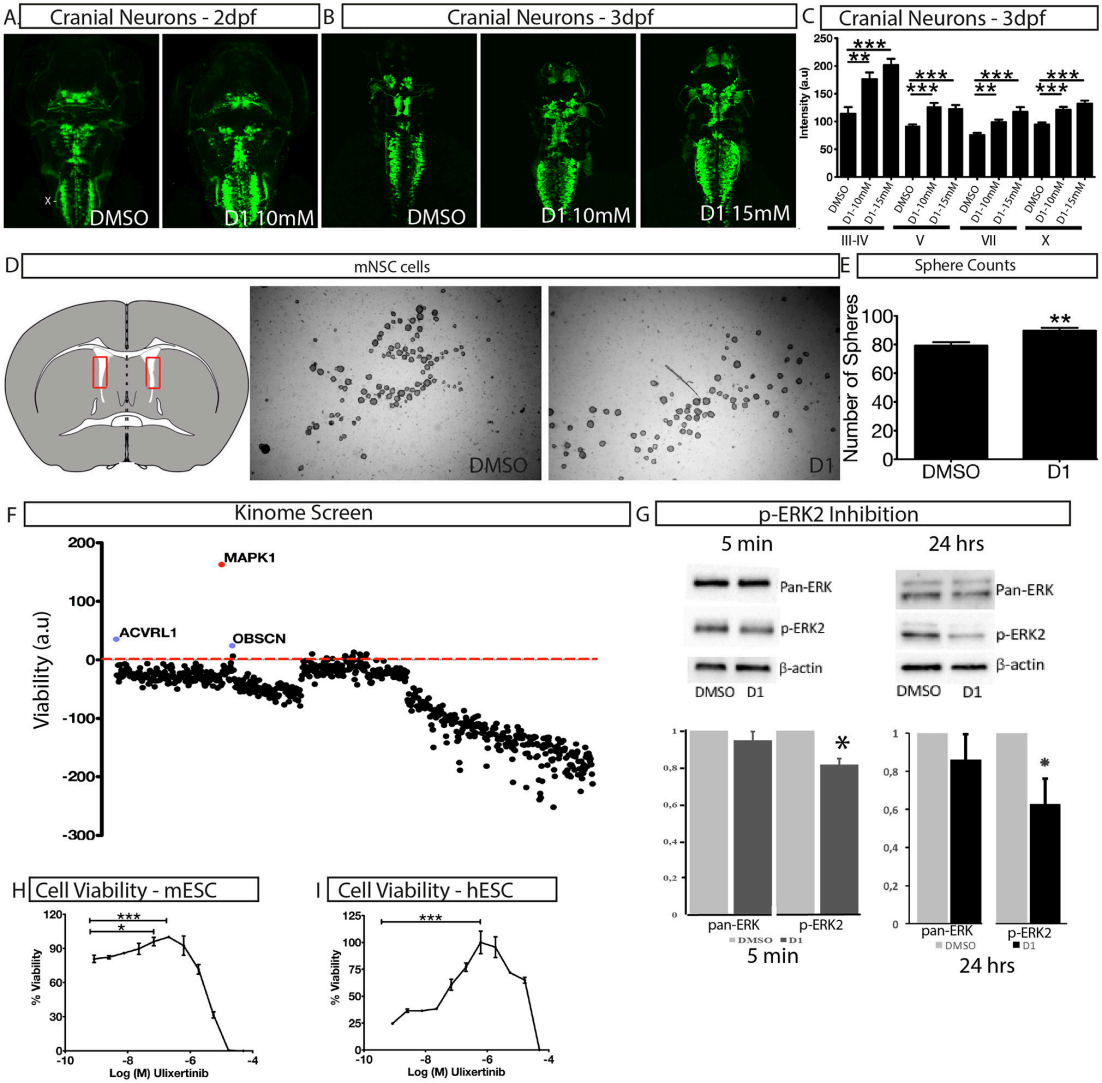
Characterization of *in vivo* and *in vitro* effect of D1. **(A,B)** The effect of D1 on cranial neuronal clusters III-IV, V, VII, and X is shown in a dorsal view on islet1:GFP transgenic larvae after 2 days **(A)** and 3 days exposure **(B,C)** fluorescence intensity of cranial neurons from islet1:GFP transgenic Zebrafish larvae after 3 days of exposure to D1. **(D)** Mouse lateral ventricle isolation (shown in the representative image as a red rectangle on the transverse brain section) and cultures to obtain neurospheres. **(E)** Quantification of the number of spheres obtained post-culture with DMSO or D1. **(F)** Kinome screen showing relative change of viability in D1 treated cell when compared to DMSO post-siRNA knockdown of all kinases. **(G)** Western blotting image and quantification of it showing inhibition of phosphorylation of ERK2 post 5 min and 24 h of D1 treatment. H-I Cell viability measurement post 4-day treatment with ERK1/2 specific inhibitor on mESC **(H)** and hESC **(I)**. mESCs, mouse embryonic stem cells; hESCs, human embryonic stem cells; mNSC, mouse neural stem cells. Data represent mean ± std, ^*^P < 0.05, ^**^P < 0.01, ^***^P < 0.001 compared to control treatment.

To assess the effect of D1 on stem cell proliferation *in vivo*, we performed a phenotype-based evaluation of D1 in two contexts, i.e., its embryonic effect and in adult neural stem cell primary culture. The embryonic effect was assessed using Islet1:GFP transgenic zebrafish expressing GFP in all cranial neurons (Higashijima et al., 2000). Zebrafish embryos were exposed to D1 at the one-cell stage and the effect on cranial motor neuron development was quantified at 2 and 3 days post-fertilization (dpf). Treatment with 10 and 15 μM D1 resulted in an increase in motor neurons that was reflected by the quantification of fluorescence intensity (Figures 3A–C). In addition to GFP intensity, many embryos also showed a clear expansion of areas characterized by different cranial neuronal clusters (Figures 3A,B). Quantification of GFP in motor neuron clusters III, IV, V, VII, and X showed a concentration dependent increase in intensity (Figure 3C). Mouse neural stem cells (mNSC) primary cultures exposed to D1 generated a more spheres (Figures 3D,E) when compared to untreated controls. The neurospheres obtained from these cultures showed expression of pluripotent markers as seen with controls (Supplementary Figure 2E). These results indicate that D1 has similar effects on proliferation *in vivo* and in stem cell primary culture derived from lateral ventricle from adult mouse. In conclusion, our combinatorial PDD screening strategy identifies D1 that increases stem cell proliferation *in vitro* and *in vivo*. Collectively, these results reinforce the strength of the PDD based screening approach in identifying novel compounds that have higher biomedical relevance.

### Mechanism of action studies

A siRNA based kinome screen was performed to understand the mechanism of action of D1 (Figure 3F). By employing a rational, that if a kinase is a key component of signal transduction triggered by D1, then, by reducing kinase activity to low levels, transiently, followed by D1 treatment would potentiate a pronounced effect on proliferation. The partial knockdown ensures that other cellular functions governed by the kinases are not dramatically altered and in time the kinase activity is restored. The screen clearly identified ERK2 (MAPK1) as potential component of D1 signaling (Figure 3F) with its partial knockdown producing an increase in viability in the presence of D1 (Figure 3F). Treatment of mESC with D1 for 5 min or 24 h showed a decrease in phosphorylation of ERK2 (Figure 3G). These results indicate that D1 treatment cause an immediate and continued inhibition of ERK2 phosphorylation in mESC cells. Furthermore, a 4-day pharmacological treatment with Ulixertinib, a known ERK1/2 specific inhibitor, produced increase in viability in both mESC and hESC cells similar to that seen with D1 treatment (Figures 3H,I).

## Discussion

One of the major drawbacks of *in vitro* phenotypic screening is the consistency of the observed phenotype across similar *in vitro* models and its translation to an *in vivo* setting. We overcame this by an integrative approach using mESCs and hESCs. A simple colony size based assay in combination with a zebrafish *in vivo* model identified a compound that promotes stem cell proliferation. The phenotype of increased colony size was accompanied by an increase of ATP corresponding to the increase in cell number which was also reflected by EdU/BrdU labeling. Therefore, the assays employed link cell proliferation to the observed phenotype. Hence, the mESC colony size phenotype based scoring approach can be used to conduct larger screens in order to identify compounds increasing proliferation of embryonic stem cells derived from human or mouse. A parallel *in vivo* screen using bright field and fluorescence imaging of zebrafish embryos allowed to evaluate the *in vitro* effect *in vivo* and allowed for toxicity analysis. Zebrafish screen using a transgenic islet1:GFP model has previously been used to assess the effect of compounds increasing stem cell proliferation on neurogenesis (Theofilopoulos et al., 2011). Therefore, this phenotypic screen provides a strict filter to identify hits that are reproducible in both *in vitro* and *in vivo* models. In conclusion, combinatorial *in vitro* and *in vivo* models identified D1 as a promising hit that increased stem cell proliferation. The same combinatorial approach was used to evaluate concentration specific effects on colony size, proliferation, viability, cytotoxicity and pluripotency.

The absence of G_0_ phase and a short G_1_ phase provide mESCs with an extraordinary capacity to undergo unlimited proliferation within a relatively short cell cycle time of 8–12 h (Savatier et al., 1994; Burdon et al., 2002; Orford and Scadden, 2008; Boheler, 2009). In addition, mESCs have additional mechanisms that control their proliferation in comparison with somatic cells. Examples are the modulation of proliferation via ion fluxes, the DNA damage checkpoint protein machinery (Andang et al., 2008) or via nucleolar proteins (Tsai and McKay, 2002; Kafienah et al., 2006).

Chemical biology based lead identification is often followed by the evaluation of compound structure activity relationships. Here, we performed SAR analysis using two different approaches, phenotypic and cell viability measurements. Each evaluation procedure has its own unique advantages: The phenotypic approach measures colony size as an indicator of cell growth and cytotoxicity. In addition, the phenotypic approach also allows us to take various biophysical parameters into account, such as colony shape, cell adhesion, spatial distribution and migration, which would not be accounted for if the SAR studies were done using biochemical assays. Cell viability measurements, such as those based on the determination of the amount of ATP in cell lysates or those based on measuring dehydrogenase activities, usually do not discriminate whether the data obtained reflect the influences of a compound on cell metabolism or instead on proliferation and therefore on the number of cells. Compound D1 produces large mESC colonies at low concentrations, which is well reflected by the amount of ATP determined, by cell counts and by EdU labeling. Higher concentrations of D1 produced small or no mESC colonies and displayed high cytotoxicity. Combination of both phenotypic and biochemical assay measurements allowed SAR evaluation in the context of cell proliferation. Phenotype and viability based SAR analysis identified compound B4 with a similar increase in colony size and ATP levels at a broader range of concentrations when compared to D1. This approach also demonstrated that structural modifications such as the substitution of the phenylamine group (R6) with indoline, nitro or piperidine and replacing the *N*-phenyl ring with cyclic or acyclic alkyl groups as seen in class I, II and III resulted in smaller or no colonies when compared to substitution of phenylamine (R6) with hydrogen or halo and substituting R4, R5, and R7 with other groups such as halo, alkyl, ester, alkoxyl or heterocycles, which resulted in a reduction of toxicity. These results identified that phenylamine (R6) and Cl (R1) groups on D1 are crucial and replacing phenylamine (R6) with tetrahydropyranylamine and acetylamine (compounds B4–B6) would lead to decreased toxicity and an increase in viability in a concentration dependent manner.

To discover the *in vivo* efficacy of D1, we undertook a broad phenotypic analysis using various models. This broad approach allowed the evaluation of D1 in the context of various areas of possible biomedical applications. Zebrafish embryos were used to evaluate neuro-developmental effects. Exposure to D1 did not reveal visible toxicity in zebrafish embryos. In zebrafish, islet1:GFP transgenic embryos express GFP in several cranial and spinal motor neurons (Higashijima et al., 2000). The generation of cranial motor neurons is a tightly controlled process wherein a pool of stem cells at the ventral midline gives rise to motor neurons that then migrate and form various cranial nuclei at different defined locations in the developing embryo (Zannino and Appel, 2009; Ravanelli and Appel, 2015). Post-differentiation, all cranial motor neurons express the islet1 transcription factor, and hence, islet1:GFP transgenic zebrafish has been effectively used in various chemical biology programs to study the relationship between stem cell proliferation and neurogenesis (Theofilopoulos et al., 2011, 2014). Similar to the effects seen in primary screening, zebrafish embryos exposed to D1 showed an increase in GFP expression in a concentration dependent manner. Mouse neural stem cells (mNSC) primary cultures exposed to D1 generated more neurospheres without any changes to their pluripotency. These results emphasize three important aspects. Firstly, the designed phenotypic screen demonstrates a similar effect of D1 on proliferation *in vivo* and in various *in vitro* models. The second aspect is the demonstration of the translatability of effects to various stem cells and the third aspect covers the biomedical relevance of the approach for the evaluation of a drug's efficacy with regard to a multitude of diseases.

The extracellular signal-regulated Mek/Erk kinase is required for cell cycle progression and proliferation of stem cells (Chang and Karin, 2001; Pearson et al., 2001; Shaul and Seger, 2007). Various chemically defined medium include Mek/Erk signaling inhibitors to facilitate ES cell derivation, maintenance of naive pluripotency of both human and mouse ESC (Hanna et al., 2010; Chan et al., 2013; Gafni et al., 2013; Takashima et al., 2014; Theunissen et al., 2014) and somatic cell reprogramming (Burdon et al., 1999; Yao et al., 2003; Li et al., 2008, 2009; Ying et al., 2008; Fang et al., 2014). Erk signaling is required for self-renewal and proliferation of mouse ESC (Guo et al., 2013). While complete loss of Erk signaling via genetic ablation of it reduces proliferation, induces apoptosis and genomic instability, partial inhibition via pharamacological inhibitors of Mek/Erk signaling promotes self-renewal of mESC (Chen et al., 2015). These studies indicate a dual role of Erk signaling in mESC, while a minimal level of signaling facilitates proliferation and cell cycle progression, expression of Erk over a certain threshold activates differentiation genes and suppresses pluripotency. Although treatment with D1 produced a significant inhibition of Erk2 phosphorylation immediately upon compound administration, a complete inhibition was not seen in either short or long term treatment. This could be due to a possible negative feedback regulation that results in some p-ERK activity. This limited p-ERK activity might be enough to promote self-renewal and proliferation of ESC while maintaining pluripotency. In addition, inhibition of Mek/Erk signaling or knockout of the upstream activators of Erk signaling interferes with lineage commitment and proper embryoid body generation. We see that human ESC treated with D1 can generate embryoid bodies as that seen in control conditions, indicating that although D1 promotes proliferation of ESC, it does not interfere in the normal differentiation process. Additional components of this signaling cascade and how modulation of Erk signaling by D1 is done in order to maintain pluripotency and proliferation remains to be explored.

In conclusion, by employing an integrative approach using mESCs, hESCs and zebrafish based PDD setup we have identified a small molecule promoting stem cell proliferation. This approach allows us to overcome a major drawback associated with PDD screening, i.e., consistency of the observed phenotype across similar *in vitro* models and its translation to an *in vivo* setting. Stem cells have additional mechanisms that control their proliferation in comparison to somatic cells (Andang et al., 2008; Chowdhury et al., 2010; Higuchi et al., 2011; Zoldan et al., 2011; Lam and Longaker, 2012; Dado-Rosenfeld et al., 2015). We see here that D1 might employ one or more such mechanisms producing its effect on proliferation across various *in vitro* and *in vivo* PDD models. We here demonstrate the robustness of the phenotypic based approach in identifying and characterizing a lead compound.

## Ethics statement

All animal works were performed in accordance with the national guidelines and Institutional guidelines. The study protocols were verified and approved by the ethical committee. The use of zebrafish and the study has been approved by the Stockholm North Animal Committee, Dnr N122/15. Rodent study was approved by ethical committee number IAEC/ERI/LC/03/17 and IAEC NO 03/2015(A).

## Author contributions

SK designed the work. CY and TF performed most of the experiments. GC, MAE, VZ, SA, AR, JJ, and FV contributed to some experiments. JM, JL, OL, OH, FG, AG, and MAN provided the material supporting and participated in writing the manuscript. SK and CY analyzed the data and edited and finalized the manuscript.

### Conflict of interest statement

The authors declare that the research was conducted in the absence of any commercial or financial relationships that could be construed as a potential conflict of interest.

## Supplementary material

Supplementary Figure 1 Phenotypic screening and cell viability assays using mESCs and hESCs and GFP expression controlled by the islet1 promoter in transgenic zebrafish. **(A)** Colony morphology based screening of mESCs and the different phenotype obtained, classified as control (DMSO treated and compounds similar to control), phenotype 1 (dead cells), phenotype 2 (small colonies), phenotype 3 (small and large colonies) and phenotype 4 (large colonies when compared to control). **(B)** Cell viability primary screening of hESCs treated with compounds producing phenotype 3 and 4 from the mESC screen. Baseline control signal is shown with a red line and compounds producing an increase in ATP are labeled with a red asterisk. All compounds are numerically labeled and the hit compound D1 is shown with an arrow mark. **(C)** Cell viability based re-screening of hESCs treated with the compounds identified (with asterisk) in the primary screen. A red line represents the baseline signal and the identified hit D1 is represented by an arrow. **(D)** Cell viability measurement of D1 treated fibroblast cells after 4-day treatment. **(E)** Representative images of zebrafish based screening, both brightfield and GFP photographs were obtained. Brightfield imaging identified compounds that did not produce developmental defects and compounds that caused developmental delay or toxicity. Fluorescence imaging identified compounds that produced an increase in fluorescence when compared to control. **(F)** Quantification of fluorescence of embryos treated with all compounds identified hit D1 as increasing fluorescence when compared to control. Abbreviations: mNSCs; mouse neural stem cells. Data represent mean ± std, ^*^*P* < 0.05, ^**^*P* < 0.01 compared to control treatment.

Supplementary Figure 2 Evaluating the effect of D1 on mouse and human embryonic stem cells. **(A)** Bright field image of colony morphology of mES cells treated with 0.05 μM D1 compared to control (DMSO). **(B)** Three different experiments showing the effect on the cell cycle profile of mESCs treated for 4 days with 0.05 μM D1 or DMSO. **(C)** Percent BrdU positive cells post-treatment with 0.05 μM D1 or DMSO for 4 days. **(D)** Immunostaining with pluripotency markers after treatment of hESC for 4 days with DMSO or 0.05 μM D1. **(E)** Immunostaining with pluripotency markers after treatment of mNSCs in primary culture for 4 days with DMSO or 0.05 μM D1. **(F)** Immunostaining with active cleaved caspase 3 antibody using mESCs after treatment for 4 days with DMSO or 0.05 μM D1. **(G)** Embryoid body generated in the presence or absence of D1. **(H)** Immunostaining of embryoid bodies post-treatment with DMSO or D1.

Supplementary Table 1 Plate ID and NSC number of hits identified in primary screening.

## References

[B1] Andang M. Hjerling-Leffler J. Moliner A. Lundgren T. K. Castelo-Branco G. Nanou E.. (2008). Histone H2AX-dependent GABA(A) receptor regulation of stem cell proliferation. Nature 451, 460–464. 10.1038/nature06488 18185516

[B2] Boheler K. R. (2009). Stem cell pluripotency: a cellular trait that depends on transcription factors, chromatin state and a checkpoint deficient cell cycle. J. Cell. Physiol. 221, 10–17. 10.1002/jcp.21866 19562686 PMC3326661

[B3] Burdon T. Smith A. Savatier P. (2002). Signalling, cell cycle and pluripotency in embryonic stem cells. Trends Cell Biol. 12, 432–438. 10.1016/S0962-8924(02)02352-8 12220864

[B4] Burdon T. Stracey C. Chambers I. Nichols J. Smith A. (1999). Suppression of SHP-2 and ERK signalling promotes self-renewal of mouse embryonic stem cells. Dev. Biol. 210, 30–43. 10.1006/dbio.1999.9265 10364425

[B5] Chan Y. S. Goke J. Ng J. H. Lu X. Gonzales K. A. Tan C. P.. (2013). Induction of a human pluripotent state with distinct regulatory circuitry that resembles preimplantation epiblast. Cell Stem Cell 13, 663–675. 10.1016/j.stem.2013.11.015 24315441

[B6] Chang L. Karin M. (2001). Mammalian MAP kinase signalling cascades. Nature 410, 37–40. 10.1038/35065000 11242034

[B7] Chen H. Guo R. Zhang Q. Guo H. Yang M. Wu Z.. (2015). Erk signaling is indispensable for genomic stability and self-renewal of mouse embryonic stem cells. Proc. Natl. Acad. Sci. U.S.A. 112, E5936–E5943. 10.1073/pnas.1516319112 26483458 PMC4640739

[B8] Chowdhury F. Li Y. Poh Y. C. Yokohama-Tamaki T. Wang N. Tanaka T. S. (2010). Soft substrates promote homogeneous self-renewal of embryonic stem cells via downregulating cell-matrix tractions. PLoS ONE 5:e15655. 10.1371/journal.pone.0015655 21179449 PMC3001487

[B9] Dado-Rosenfeld D. Tzchori I. Fine A. Chen-Konak L. Levenberg S. (2015). Tensile forces applied on a cell-embedded three-dimensional scaffold can direct early differentiation of embryonic stem cells toward the mesoderm germ layer. Tissue Eng. A 21, 124–133. 10.1089/ten.tea.2014.0008 25002337 PMC4293137

[B10] Deferrari G. Ravera M. Berruti V. (2003). Treatment of diabetic nephropathy in its early stages. Diabetes Metab. Res. Rev. 19, 101–114. 10.1002/dmrr.363 12673778

[B11] Fang R. Liu K. Zhao Y. Li H. Zhu D. Du Y.. (2014). Generation of naive induced pluripotent stem cells from rhesus monkey fibroblasts. Cell Stem Cell 15, 488–496. 10.1016/j.stem.2014.09.004 25280221

[B12] Fu X. Toh W. S. Liu H. Lu K. Li M. Cao T. (2011). Establishment of clinically compliant human embryonic stem cells in an autologous feeder-free system. Tissue Eng. C Methods 17, 927–937. 10.1089/ten.tec.2010.0735 21561302

[B13] Gafni O. Weinberger L. Mansour A. A. Manor Y. S. Chomsky E. Ben-Yosef D.. (2013). Derivation of novel human ground state naive pluripotent stem cells. Nature 504, 282–286. 10.1038/nature12745 24172903

[B14] Guo W. Hao B. Wang Q. Lu Y. Yue J. (2013). Requirement of B-Raf, C-Raf, and A-Raf for the growth and survival of mouse embryonic stem cells. Exp. Cell Res. 319, 2801–2811. 10.1016/j.yexcr.2013.09.006 24051329

[B15] Hanna J. Cheng A. W. Saha K. Kim J. Lengner C. J. Soldner F.. (2010). Human embryonic stem cells with biological and epigenetic characteristics similar to those of mouse ESCs. Proc. Natl. Acad. Sci. U.S.A. 107, 9222–9227. 10.1073/pnas.1004584107 20442331 PMC2889088

[B16] Higashijima S. Hotta Y. Okamoto H. (2000). Visualization of cranial motor neurons in live transgenic zebrafish expressing green fluorescent protein under the control of the islet-1 promoter/enhancer. J. Neurosci. 20, 206–218. 10627598 10.1523/JNEUROSCI.20-01-00206.2000 PMC6774115

[B17] Higuchi A. Ling Q. D. Ko Y. A. Chang Y. Umezawa A. (2011). Biomaterials for the feeder-free culture of human embryonic stem cells and induced pluripotent stem cells. Chem. Rev. 111, 3021–3035. 10.1021/cr1003612 21344932

[B18] Johansson H. Svensson F. Runnberg R. Simonsson T. Simonsson S. (2010). Phosphorylated nucleolin interacts with translationally controlled tumor protein during mitosis and with Oct4 during interphase in ES cells. PLoS ONE 5:e13678. 10.1371/journal.pone.0013678 21048921 PMC2965110

[B19] Kafienah W. Mistry S. Williams C. Hollander A. P. (2006). Nucleostemin is a marker of proliferating stromal stem cells in adult human bone marrow. Stem Cells 24, 1113–1120. 10.1634/stemcells.2005-0416 16282439

[B20] Kimmel C. B. Ballard W. W. Kimmel S. R. Ullmann B. Schilling T. F. (1995). Stages of embryonic development of the zebrafish. Dev. Dyn. 203, 253–310. 10.1002/aja.1002030302 8589427

[B21] Kitambi S. S. Chandrasekar G. (2011). Stem cells: a model for screening, discovery and development of drugs. Drug Des. Dev. Ther. 10, 2881–2897. 10.2147/SCCAA.S16417 PMC3781757 24198530

[B22] Lam M. T. Longaker M. T. (2012). Comparison of several attachment methods for human iPS, embryonic and adipose-derived stem cells for tissue engineering. J. Tissue Eng. Regen. Med. 6(Suppl. 3), s80–s86. 10.1002/term.1499 22610948 PMC4086291

[B23] Lee J. A. Berg E. L. (2013). Neoclassic drug discovery: the case for lead generation using phenotypic and functional approaches. J. Biomol. Screen. 18, 1143–1155. 10.1177/1087057113506118 24080259

[B24] Lee J. A. Uhlik M. T. Moxham C. M. Tomandl D. Sall D. J. (2012). Modern phenotypic drug discovery is a viable, neoclassic pharma strategy. J. Med. Chem. 55, 4527–4538. 10.1021/jm201649s 22409666

[B25] Li P. Tong C. Mehrian-Shai R. Jia L. Wu N. Yan Y.. (2008). Germline competent embryonic stem cells derived from rat blastocysts. Cell 135, 1299–1310. 10.1016/j.cell.2008.12.006 19109898 PMC2735113

[B26] Li W. Wei W. Zhu S. Zhu J. Shi Y. Lin T.. (2009). Generation of rat and human induced pluripotent stem cells by combining genetic reprogramming and chemical inhibitors. Cell Stem Cell 4, 16–19. 10.1016/j.stem.2008.11.014 19097958

[B27] Orford K. W. Scadden D. T. (2008). Deconstructing stem cell self-renewal: genetic insights into cell-cycle regulation. Nat. Rev. Genet. 9, 115–128. 10.1038/nrg2269 18202695

[B28] Pearson G. Robinson F. Beers Gibson T. Xu B. E. Karandikar M. Berman K.. (2001). Mitogen-activated protein (MAP) kinase pathways: regulation and physiological functions. Endocr. Rev. 22, 153–183. 10.1210/er.22.2.153 11294822

[B29] Phiel C. J. Wilson C. A. Lee V. M. Klein P. S. (2003). GSK-3alpha regulates production of Alzheimer's disease amyloid-beta peptides. Nature 423, 435–439. 10.1038/nature01640/nature01640 12761548

[B30] Ravanelli A. M. Appel B. (2015). Motor neurons and oligodendrocytes arise from distinct cell lineages by progenitor recruitment. Genes Dev. 29, 2504–2515. 10.1101/gad.271312.115 26584621 PMC4691953

[B31] Rodin S. Antonsson L. Hovatta O. Tryggvason K. (2014). Monolayer culturing and cloning of human pluripotent stem cells on laminin-521—based matrices under xeno-free and chemically defined conditions. Nat. Protoc. 9, 2354–2368. 10.1038/nprot.2014.159 25211513

[B32] Sams-Dodd F. (2005). Target-based drug discovery: is something wrong? Drug Discov. Today 10, 139–147. 10.1016/s1359-6446(04)03316-1 15718163

[B33] Sams-Dodd F. (2013). Is poor research the cause of the declining productivity of the pharmaceutical industry? An industry in need of a paradigm shift. Drug Discov. Today 18, 211–217. 10.1016/j.drudis.2012.10.010 23131208

[B34] Savatier P. Huang S. Szekely L. Wiman K. G. Samarut J. (1994). Contrasting patterns of retinoblastoma protein expression in mouse embryonic stem cells and embryonic fibroblasts. Oncogene 9, 809–818. 8108123

[B35] Shaul Y. D. Seger R. (2007). The MEK/ERK cascade: from signaling specificity to diverse functions. Biochim. Biophys. Acta 1773, 1213–1226. 10.1016/j.bbamcr.2006.10.005 17112607

[B36] Sievertzon M. Wirta V. Mercer A. Frisen J. Lundeberg J. (2005). Epidermal growth factor (EGF) withdrawal masks gene expression differences in the study of pituitary adenylate cyclase-activating polypeptide (PACAP) activation of primary neural stem cell proliferation. BMC Neurosci. 6:55. 10.1186/1471-2202-6-55 16124881 PMC1208901

[B37] Swinney D. C. Anthony J. (2011). How were new medicines discovered? Nat. Rev. Drug Discov. 10, 507–519. 10.1038/nrd3480 21701501

[B38] Szabo M. Akusjärvi S. S. Saxena A. Liu J. Chandrasekar G. Kitambi S. S. (2017). Cell and small animal models for phenotypic drug discovery. Drug Des. Dev. Ther. 11, 1957–1967. 10.2147/DDDT.S129447 28721015 PMC5500539

[B39] Takashima Y. Guo G. Loos R. Nichols J. Ficz G. Krueger F.. (2014). Resetting transcription factor control circuitry toward ground-state pluripotency in human. Cell 158, 1254–1269. 10.1016/j.cell.2014.08.029 25215486 PMC4162745

[B40] Theofilopoulos S. Griffiths W. J. Crick P. J. Yang S. Meljon A. Ogundare M.. (2014). Cholestenoic acids regulate motor neuron survival via liver X receptors. J. Clin. Invest. 124, 4829–4842. 10.1172/JCI68506 25271621 PMC4347238

[B41] Theofilopoulos S. Karu K. Kitambi S. Sacchetti P. Sousa K. Sjovall J. (2011). Identification and characterisation of endogenous LXR ligands in ventral midbrain development. Neurosci. Res. 71:E50 10.1016/j.neures.2011.07.211

[B42] Theofilopoulos S. Wang Y. Kitambi S. S. Sacchetti P. Sousa K. M. Bodin K.. (2013). Brain endogenous liver X receptor ligands selectively promote midbrain neurogenesis. Nat. Chem. Biol. 9, 126–133. 10.1038/nchembio.1156 23292650

[B43] Theunissen T. W. Powell B. E. Wang H. Mitalipova M. Faddah D. A. Reddy J. (2014). Systematic identification of culture conditions for induction and maintenance of naive human pluripotency. Cell Stem Cell 15, 471–487. 10.1016/j.stem.2014.07.002 25090446 PMC4184977

[B44] Tsai R. Y. McKay R. D. (2002). A nucleolar mechanism controlling cell proliferation in stem cells and cancer cells. Genes Dev. 16, 2991–3003. 10.1101/gad.55671 12464630 PMC187487

[B45] Wachs F. P. Couillard-Despres S. Engelhardt M. Wilhelm D. Ploetz S. Vroemen M.. (2003). High efficacy of clonal growth and expansion of adult neural stem cells. Lab. Invest. 83, 949–962. 10.1097/01.LAB.0000075556.74231.A5 12861035

[B46] Yang A. Shi G. Zhou C. Lu R. Li H. Sun L.. (2011). Nucleolin maintains embryonic stem cell self-renewal by suppression of p53 protein-dependent pathway. J. Biol. Chem. 286, 43370–43382. 10.1074/jbc.M111.225185 22013067 PMC3234871

[B47] Yao Y. Li W. Wu J. Germann U. A. Su M. S. Kuida K.. (2003). Extracellular signal-regulated kinase 2 is necessary for mesoderm differentiation. Proc. Natl. Acad. Sci. U.S.A. 100, 12759–12764. 10.1073/pnas.2134254100 14566055 PMC240691

[B48] Ying Q. L. Wray J. Nichols J. Batlle-Morera L. Doble B. Woodgett J.. (2008). The ground state of embryonic stem cell self-renewal. Nature 453, 519–523. 10.1038/nature06968 18497825 PMC5328678

[B49] Zannino D. A. Appel B. (2009). Olig2+ precursors produce abducens motor neurons and oligodendrocytes in the zebrafish hindbrain. J. Neurosci. 29, 2322–2333. 10.1523/JNEUROSCI.3755-08.2009 19244509 PMC2720165

[B50] Zoldan J. Karagiannis E. D. Lee C. Y. Anderson D. G. Langer R. Levenberg S. (2011). The influence of scaffold elasticity on germ layer specification of human embryonic stem cells. Biomaterials 32, 9612–9621. 10.1016/j.biomaterials.2011.09.012 21963156 PMC3313669

