# Supplementary figures and images for: Phenotypic Screen Identifies a Small Molecule Modulating ERK2 and Promoting Stem Cell Proliferation
Source: Front Pharmacol. 2017 Oct 24;8:726. doi: 10.3389/fphar.2017.00726 (PMC5660848; doi:10.3389/fphar.2017.00726)

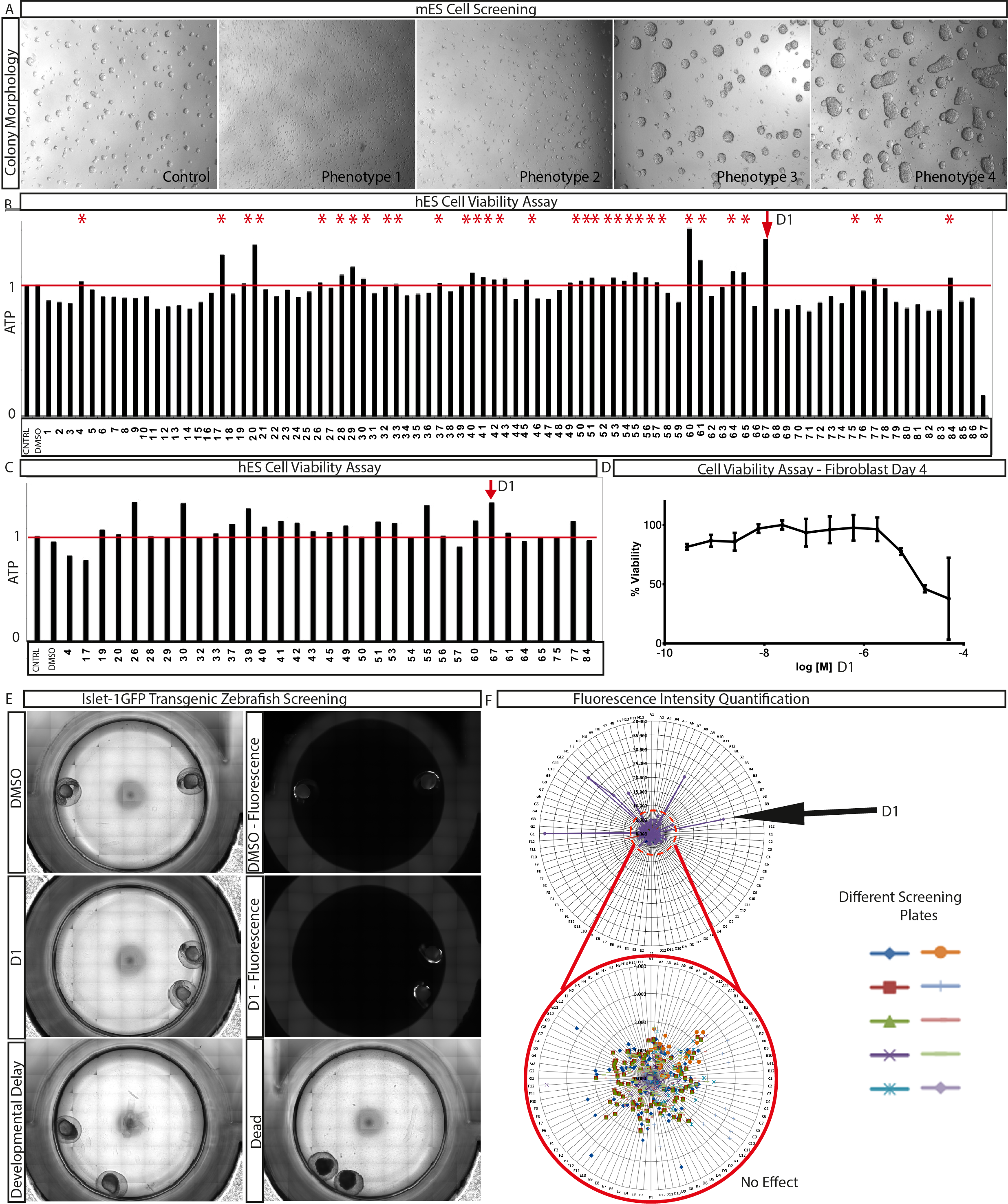

Supplement: Supplementary Figure 1 — Phenotypic screening and cell viability assays using mESCs and hESCs and GFP expression controlled by the islet1 promoter in transgenic zebrafish. (A) Colony morphology based screening of mESCs and the different phenotype obtained, classified as control (DMSO treated and compounds similar to control), phenotype 1 (dead cells), phenotype 2 (small colonies), phenotype 3 (small and large colonies) and phenotype 4 (large colonies when compared to control). (B) Cell viability primary screening of hESCs treated with compounds producing phenotype 3 and 4 from the mESC screen. Baseline control signal is shown with a red line and compounds producing an increase in ATP are labeled with a red asterisk. All compounds are numerically labeled and the hit compound D1 is shown with an arrow mark. (C) Cell viability based re-screening of hESCs treated with the compounds identified (with asterisk) in the primary screen. A red line represents the baseline signal and the identified hit D1 is represented by an arrow. (D) Cell viability measurement of D1 treated fibroblast cells after 4-day treatment. (E) Representative images of zebrafish based screening, both brightfield and GFP photographs were obtained. Brightfield imaging identified compounds that did not produce developmental defects and compounds that caused developmental delay or toxicity. Fluorescence imaging identified compounds that produced an increase in fluorescence when compared to control. (F) Quantification of fluorescence of embryos treated with all compounds identified hit D1 as increasing fluorescence when compared to control. Abbreviations: mNSCs; mouse neural stem cells. Data represent mean ± std, *P < 0.05, **P < 0.01 compared to control treatment. [file Image1.TIF]

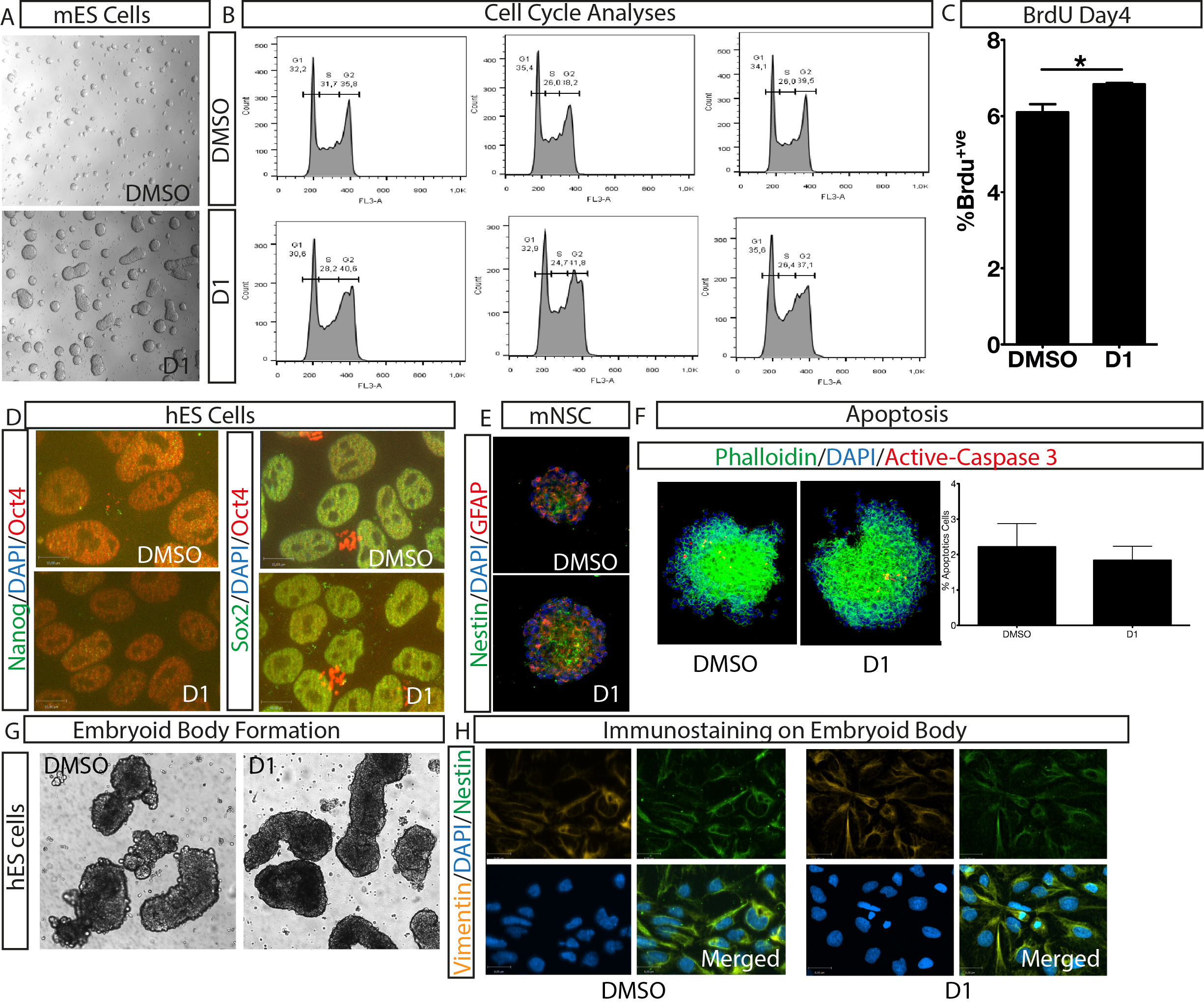

Supplement: Supplementary Figure 2 — Evaluating the effect of D1 on mouse and human embryonic stem cells. (A) Bright field image of colony morphology of mES cells treated with 0.05 μM D1 compared to control (DMSO). (B) Three different experiments showing the effect on the cell cycle profile of mESCs treated for 4 days with 0.05 μM D1 or DMSO. (C) Percent BrdU positive cells post-treatment with 0.05 μM D1 or DMSO for 4 days. (D) Immunostaining with pluripotency markers after treatment of hESC for 4 days with DMSO or 0.05 μM D1. (E) Immunostaining with pluripotency markers after treatment of mNSCs in primary culture for 4 days with DMSO or 0.05 μM D1. (F) Immunostaining with active cleaved caspase 3 antibody using mESCs after treatment for 4 days with DMSO or 0.05 μM D1. (G) Embryoid body generated in the presence or absence of D1. (H) Immunostaining of embryoid bodies post-treatment with DMSO or D1. [file Image2.TIF]
